# 4-chlorophenol removal by air lift packed bed bioreactor and its modeling by kinetics and numerical model (artificial neural network)

**DOI:** 10.1038/s41598-020-79968-7

**Published:** 2021-01-12

**Authors:** Elahe Azizi, Fariba Abbasi, Mohammad Ali Baghapour, Mohammad Reza Shirdareh, Mohammad Reza Shooshtarian

**Affiliations:** Department of Environmental Health Engineering, Shiraz University of Medical Science, Shiraz, Iran

**Keywords:** Environmental sciences, Natural hazards

## Abstract

4-chlorophenol (4-CP) is a hazardous contaminant that is hardly removed by some technologies. This study investigated the biodegradation, and physical 4-CP removal by a mixed microbial consortium in the Airlift packed bed bioreactor (ALPBB) and modeling by an artificial neural network (ANN) for first the time. The removal efficiency of ALPBB was investigated at 4-CP(1-1000 mg/L) and hydraulic retention time (HRT)(6-96 hr) by HPLC. The results showed that removal efficiency decreased from 85 at 1 to 0.03% at 1000 mg/L, with increasing 4-CP concentration and HRT decreasing. BOD_5_/COD increased with increasing exposure time and concentration decreasing, from 0.05 at 1000 to 0.96 at 1 mg/L. With time increasing, the correlation between COD and 4-CP removal increased (R^2^ = 0.5, HRT = 96 h). There was a positive correlation between the removal of 4-CP and SCOD by curve fitting was R^2^ = 0.93 and 0.96, respectively. Moreover, the kinetics of 4-CP removal follows the first-order and pseudo-first-order equation at 1 mg/L and other concentrations, respectively. 4-CP removal modeling has shown that the 2:3:1 and 2:4:1 were the best structures (MSE: physical = 0.126 and biological = 0.9)(R^2^_all_physical = 0.999 and R^2^_test_physical = 0.999) and (R^2^_all_biological = 0.71, and R^2^_test_biological = 0.997) for 4-CP removal. Also, the output obtained by the ANN prediction of 4-CP was correlated to the actual data (R^2^_physical_ = 0.9997 and R^2^_biological_ = 0.59). Based on the results, ALPBB with up-flow submerged aeration is a suitable option for the lower concentration of 4-CP, but it had less efficiency at high concentrations. So, physical removal of 4-CP was predominant in biological treatment. Therefore, the modification of this reactor for 4-CP removal is suggested at high concentrations.

## Introduction

Humans have caused environmental pollution and disturbed ecological balance by increasing the production and use of hazardous chemicals. Some resistant chemicals, such as pesticides, are transferred through the environment by using air and water and affect the ecosystem. The presence of phenol, especially Chlorophenols, in the structure of insecticides increases their toxicity and non-degradability^1^. Chlorophenols are organic compounds that are composed of one or more chlorine atoms (at least one and at most five atoms) on a phenolic ring and can form nineteen different types of this compound^2^. The most common of these compounds are chlorophenols with a chlorine atom number of two or less. The chemical structure of 4-chlorophenol (4-CP) is a chlorine atom substituted with hydrogen in the phenol benzene ring^3^. Due to this substitution, the compound has properties^3^ that make it suitable for many industrial applications such as the preparation of pesticides, disinfectants, and wood preservatives^4^.

Subsequently, these industries enter the 4-CP to the environment through sewage^5–7^. Although the maximum permissible concentration of phenolic compounds in drinking water by the WHO is 0.001 mg/L, and the permitted level of discharge to lakes is about 0.1 mg/l^8^, the high concentration of it is entering to water body. This compound causes some environmental and health hazards and problems, including carcinogenicity, mutagenicity, cytotoxicity, corrosion, irritation of the skin and eyes, throat, nose, respiratory and other problems which are noted^4,9^. Moreover, in the classification of the European Water Pollution Control Committee due to high environmental hazards, these compounds are ranked 38 to 43 among toxic pollutants^2,10,11^. Besides, this compound can accumulate, therefore, it is difficult to degrade in the environment due to their high stability^7,12^. Therefore, 4-CP removal from the wastewater of related industries is essential^13^.

The various physical and chemical methods have been used to remove these compounds from aqueous media, including adsorption, optical decomposition, solvent extraction, or chemical oxidation^11,14–16^. Some of them, Ni_2_Pd/KCC-1 and Fe_3_O_4_/CeO_2_ have shown a high ability to remove this compound, and NiO/UV/H_2_O_2_ was able to remove 100% 4-CP^17–19^. Despite the high removal efficiency, the main limitation of these methods is the high cost and production of by-products that often require further treatment^20–22^. On the other hand, biological methods are also used to remove these compounds because species such as *Arthrobacterchlorophenolicus A6, Rhodococcusopacus 1G*, *Ralstoniapickettii LD1*, or *Pseudomonas knackmusii B-13 and Bacillus subtilis MF447840.1*. Because they use phenolic compounds as carbon and energy sources^16,30^. Moreover, with optimizing of pH, agitation, temperature, and inoculum age parameters, some species, such as *Comamonastestosteroni CECT 326 T* strain and *Arthrobacterchlorophenolicus A6*, remove the 4-CP completely^23,24^. Among biological system for treatment of 4-CP, immobilized catalyzed packed bed reactor, sequencing batch reactor (SBR) and modified sequential batch reactor (MSBR) have high efficiency (99%). Besides, the rate of biofilm accumulation and removal efficiency in the up-flow anaerobic sludge blanket (UASB) system has also been investigated^25,26,31^.

However, one of the well-known processes of wastewater treatment is biofilters^34^. That upflow air types are widely used in industrial wastewater treatment. So, this method has shown successful results in the removal of resistant organic compounds such as amoxicillin and atrazine^27,28,33^, but one of the most disadvantages of this biofilter is the physical removal of 4-CP due to media tourbalance. Because of the physical removal of 4-CP, it emits to surrounding air that is challenging for human health and air pollution. In other words, removal efficiency modeling of this reactor is a suitable method for the prediction and development of this system. There is a linear and non-linear model for the prediction of removal of contamination. Although some linear model such as the kinetics model predicts the trend of removal efficiency^32^, most of the removal system follows the non-linear model. Among the non-linear models, the ANN is a powerful method that is a numerical model, and the response is predicted based on the databases. Therefore, this study aimed to evaluate the efficiency of ALPBB with plastic media for the removal of 4-CP and equivalent soluble chemical oxygen demand (SCOD) from aqueous media. The use of plastic media is one of the advantages of this reactor that are available and inexpensive, and it is light media that is suspended in bioreactor easily. Therefore, the cost-effectiveness of operation bioreactor increase. Moreover, the physical and biological removal of 4-CP was compared using ALPBB in various contact time and concentration of 4-CP for the first time. Then the kinetics of 4-CP removal with ALPBB investigated with the first, second, and pseudo-first-order models. Finally physical and biological removal of 4-CP predicted by curve design and ANN modelling for the first time.

## Materials and method

### Materials

The used materials in this study were the analysis grade with a purity of more than 98%. The crystalline form of 4-CP (Sigma-Aldrich) was prepared and standardized with 98% purity to inject into HPLC. Ionized distilled water was used for BOD and COD experiments, and deionized water (Millipore—MilliQ) was used to prepare the mobile phase for HPLC.

### The proposed air lift packed bed bioreactor

**Figure 1:**
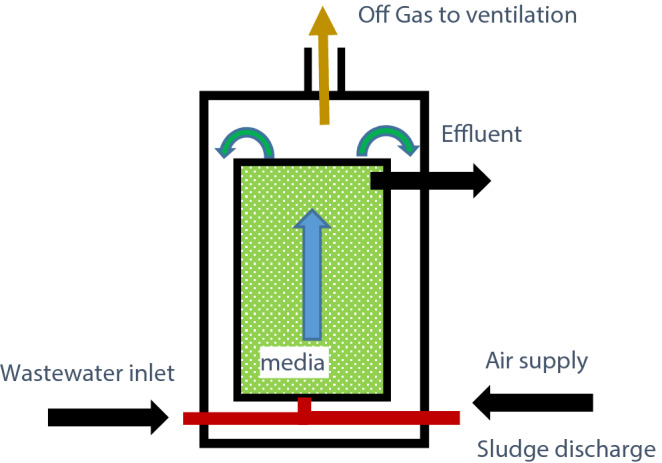
Perspective of the proposed used ALPBB.

The used ALPBB in this study was designed and operated on a pilot-scale that is shown in Fig. 1.

The reactor consists of a *Polyvinyl chloride* (PVC) column with two internal and external walls. The inner wall forms the media for removal reaction of 4-CP, which was filled with a plastic substrate. These media with specific properties (Table 1) provide sufficient surface area for attachment and growth of microorganisms. The outer wall (Table 1) also consists of a sheathed pipe embedded for sludge return to the reactor.

**Table 1:** Media and dimensional characters of the air lift packed bed bioreactor.

Character	Specifications or amounts
Type	Fixed
Shape	Wavy ring
Material	HDPE ^1^
Density	186 ± 2 (kg/m^3^)
Specific gravity	0.98
Specific surface	410 (m^2^ m^3^)
Thickness	350 (µm)
External diameter	15 (mm)
Internal diameter	12 (mm)
Height	11–13 (mm)
Porosity	90 (%)
Dimension (mm)	
Main reactor	100
External wall	110
Height (mm)	
Main reactor	600
External wall	1000
$Volume_{t}$ ^2^(lit)	
Main reactor	4.7
External wall	9.4
$Volume_{e}$ ^3^(lit)	
Main reactor	4.5
External wall	4.8

^1^High density poly ethylene.

^2^Total volume.

^3^Effective volume.

The required oxygen for the reactor was supplied from the bottom of the reactor by an air diffuser. The role of aeration was to keep the substrates submerged, provide the required oxygen for the bacteria, and hydraulically sludge return, which was done by raising the surface of the sewage in the reactor and dropping into the outer wall. As a consequence of this mechanism, it can return biomass to the reactor and provide complete mixing conditions. Alongside the main reactor, a reactor was used as a control to investigate the rate of 4-CP removal from the wastewater through the 4-CP existence from the surface, by aeration or adsorption to the bed as well as physical removal due to turbulence of reactors media.

### Synthetic wastewater

The used synthetic wastewater to feed the ALPBB was a combination of sucrose as a carbon source, ammonium chloride as a nitrogen source and potassium dihydrogen phosphate, and di-potassium hydrogen phosphate as a source of phosphorus and growth stimulants. The chemical composition of synthetic wastewater is shown in Table 2.

**Table 2:** Chemical composition of synthetic wastewater.

Type	Compound	Concentration (g/l)
Nutrient	C_6_H_12_O_6_	500–1000
	NH_4_Cl	1
	K_2_HPO_4_	0.5
	KH_2_PO_4_	0.5
Growing motivator	MgSo_4_	0.1
	MnSo_4_	0.1
	CaCl_2_·2H_2_O	0.01
	FeSO_4_·7H_2_O	0.001
	4-CP	Variable (0.001, 0.01, 0.1, 1)

Among the present compounds in wastewater, sucrose and 4-CP produce COD equivalent to 5000 mg/L.

### Microbial adaptation and setting up the air lift packed bed bioreactor

To reactor start-up, Shiraz municipal wastewater treatment sludge was used to seeding and providing a bacterial consortium. To set up the ALPBB, first, the activated sludge and synthetic wastewater added to the reactor to supply the needed food for the bacteria. After the growth of bacteria on the bed (7–14 days), the biofilm was formed to bacteria immobilize on its. Then, the wastewater in the reactor was discharged, and the synthetic wastewater was added to it (the concentration of 4-CP = 0.1 mg/L). In this stage, the bacteria use 4-CP as a source of carbon and energy. After microbial adaptation that the biologic layer reached to sufficient thickness, concentrations of 1, 10, 100, and 1000 mg/L 4-CP were added to the wastewater. The removal rate of each concentration was evaluated at HRTs of 6, 12, 24, 48 and 96 h. Reactor operation continued until the steady-state predominated in each run. The experiments and operation of the reactor took 350 days. Table 3 shows the states of reactor operation.

**Table 3:** Different statuses for air lift packed bed bioreactor operating.

$C_{i}$ ^1^(mg/l)	Run No	HRT (h)	Repetitions	OLR ^2^ (g COD/l.d)
1	1	96	3	0.0005
	2	48	3	0.0010
	3	24	5	0.0020
	4	12	6	0.0040
	5	6	6	0.0080
10	6	96	3	0.0045
	7	48	3	0.0090
	8	24	5	0.0180
	9	12	6	0.0360
	10	6	6	0.0720
100	11	96	3	0.0370
	12	48	3	0.0750
	13	24	5	0.1500
	14	12	6	0.3000
	15	6	6	0.6000
1000	16	96	3	0.3420
	17	48	3	0.6850
	18	24	5	1.3700
	19	12	6	2.7400
	20	6	6	5.4800

^1^Initial concentration of 4-CP.

^2^Organic load rate.

### Examinations

#### Examined parameters

The measured parameters in this study included residual concentrations of 4-CP in affluent, SCOD, and biological oxygen demand (BOD_5_). The first two parameters were measured to determine the ALPBB efficiency at the beginning and end of each run. BOD5 was measured to determine the BOD_5_/COD ratio and to verify the biodegradability of the effluent at the end of the treatment periods for each of the 4-CP concentrations.

#### Extraction and determination of 4-chlorophenol in the liquid phase

Each sample taken from the reactor’s outlet effluent was centrifuged after passing throughout the filter to further suspended matter removal for 10 min at a speed of 10,000 rpm. The sample was injected into *High-performance liquid chromatography* (HPLC) after passing the membrane filter. The concentration of 4-CP was performed by HPLC with a binary pump (Waters 1525) and a Dual λ absorbance detector (Waters 2487). Column type was C_18_ Reverse Phase 250 × 4.6 mm (Spherisorb, water, USA). The mobile phases were acetonitrile and water, with a volume ratio of 40:60. The flow rate of the mobile phase was 1 ml/min, and the column temperature was 25℃. The used detector was a UV detector (UV waters 2487, dual y absorbance, and wavelength = 280 nm). The sample volume injection was 20 μl. Before the injection of unknown samples into the device, standard solutions were injected to determine the location of the 4-CP peak. Specifications for the standard peak of 4-CP included concentration = 100 μg/L, start time = 11.61 min, end time = 12.89 min and retention time = 12.12 min. In addition to the main reactor, these were also done for the control reactor.

#### Extraction and determination of 4-chlorophenol in biofilm

If 4-CP enters to biofilm, it is likely to be released into the environment. For this reason, the accumulation of 4-CP in the microbial mass was investigated at the end of each run. Water and acetonitrile were then added in equal proportions and placed in an ultrasonic bath to lysis of microbial mass with sound and heat (temperature = 95 °C for 10 min and frequency = 60 Hz). After this time, the test tube was well stirred to release 4-CP into the solution. Then, the test tube was placed in non-moving conditions to precipitate biofilm. Finally, the supernatant was centrifuged and injected into the HPLC for analysis. Throughout the experiment, pH was in the range of 6–8 and temperature in the laboratory temperature range. In this study, the experiment was designed based on full factorial, and each run was repeated in triplicate.

### 4-chlorophenol and soluble chemical oxygen demand removal modeling

The removal patterns of 4-CP and SCOD were modeled using Curve Expert. Pro1.6 software. This software is a comprehensive tool for curve fitting. These models can predict the removal of 4-CP and SCOD for unknown values of concentration and retention time. In this modeling, the effect of two independent variables of *hydraulic retention time* (HRT) and the logarithm of 4-CP concentration input to the reactor and the removal efficiency of 4-CP and SCOD as dependent variables were investigated. However, the biological treatment of contaminants in water, wastewater and others attributed to nonlinear modelling. Also, artificial neural network (ANN) is a powerful nonlinear numerical method for determining the relationship between variables. In this model, the response of model determine based on input database. In this study, the removal efficiency of 4-CP modelled by using feed-forward backpropagation and MATLAB 2018. Then based on the number of input and output variables, 2–6 neurons in the hidden layer were selected based on Eqs. 1 and 2.

1 $$ \frac{{2\left( {i + o} \right)}}{3} < n < i\left( {i + o} \right) - 1 $$

2 $$ 0.5{\text{i}} - 2 < {\text{n}} < 2{\text{i}} + 2 $$

i = number of inputs, o = the number of outputs and n = number of hidden layer neuron.

Initially, the network was trained with laboratory data that 70, 15, and 15% were used for training, validation, and testing, respectively. After training, the validation error was monitored to determine the weight and bias. So that, training was stopped to prevent over-fitting when the validation error was increased by a specific iteration. Finally, two criteria of mean square error (MSE) and correlation coefficient (R) were used to determine the best ANN structure for 4-CP removal.

## Results and discussion

### Air lift packed bed bioreactor efficiency for removal of 4-chlorophenol and COD

**Figure 2:**
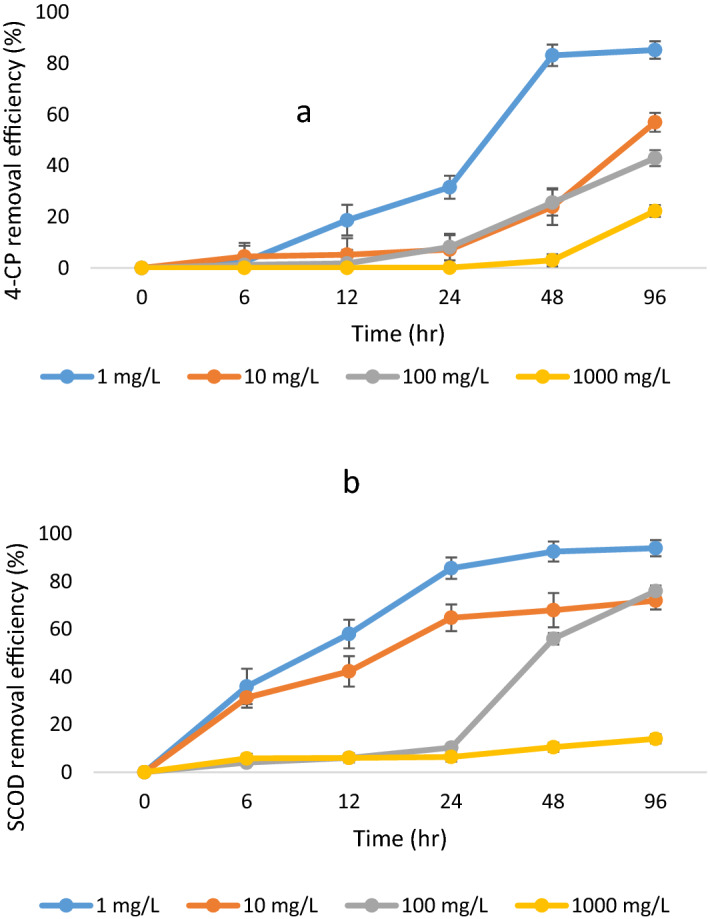
(**a**) 4-chlorophenol and (**b**) SCOD removal trends in Air lift packed bed bioreactor.

In this study, considering the importance of SCOD measurement in effluent discharge, the efficiency of ALPBB in 4-CP and COD removal at (6, 12, 24, 48, and 96 h.) HRT and 4-CP concentrations (1, 10, 100, and 1000 mg/l) were evaluated (Fig. 2).

As shown in Fig. 2, although the dependence of COD removal on contact time was less than 4-CP (P_48, 96 h_ = 0.7), the reactor removal efficiency of 4-CP and COD decreased with increasing time and decreasing 4-CP concentration. However, at concentrations of 100 and 1000 mg/L, no significant difference was observed between 4-CP and COD removal efficiency at different times (P > 0.05). So, the difference in 4-CP removal efficiency was significant between 6 and 96 h (p = 0.04) and 12.24 h (p = 0.042). As a consequence, the biological removal of 4-CP was less and inversely correlated with initial concentration (R^2^ < 0.1). Only there was a higher correlation observed at HRT = 48 h (R^2^ = 0.8), which could be due to the logarithmic growth of the microbial population. Because at the logarithmic phase, the microbial mass use of 4-CP as a carbon source. Thus, the retention time is an influential factor in the removal of low 4-CP concentrations. The results of this study showed that ALPBB at low concentrations and high retention time had an acceptable ability to SCOD removal. Accordingly, the BOD/COD ratio increased significantly at 96 h compared to 6 h (Table 4), because at low concentrations, it is possible to break 4-CP and decompose this toxic compound by a biological method such as adsorption due to the compatibility of microorganisms with the contaminant. Over time, many non-degradable compounds become biodegradable under the influence of the enzymatic process and due to the self-feeding growth of bacteria^29^. However, at high concentrations, a decrease of biofilm thickening may occur due to shock to microorganisms, bulking growth, and pink flocks of sludge.

**Table 4:** The variation of BOD_5_/COD in air lift packed bed bioreactor.

4-CP concentration (mg/l)	Organic substances in effluent					
	6 h			96 h		
	BOD_5_/COD	COD (mg/l)	BOD_5_ (mg/l)	BOD_5_/COD	COD (mg/l)	BOD_5_ (mg/l)
1	286	2600	0.11	451.2 451.2	470	0.96
10	354.3	3600	0.017	360.8	2600	0.21 0.21
100	335	3700	0.067	354.3	1500	0.28
1000	289.6	6700	0.05	295	5900	0.053

**Figure 3:**
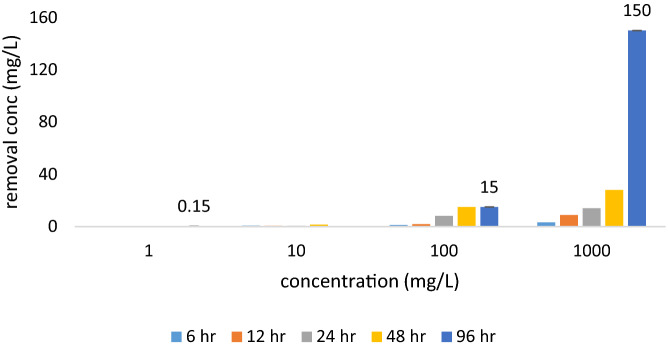
The trend of physical and biological removal of 4-chlorophenol.

In addition to, part of the contaminants trapped inside the biofilm which results has shown that the accumulation rate also increased with increasing inlet concentration. The findings of the current study show similar results with previous studies^26^. Nevertheless, some of the biofilms inside the bubbles were caused by the foam-producing bacteria. They may not participate in the pollutant removal process. These species predominate by increasing the concentration of compounds such as CaCl_2_ and K_2_HPO_4_ and decreasing of MgSo_4_^24^. Hence, in the current study, a higher correlation was observed between COD removal and 4-CP with increasing retention time (6–96 h) (r^2^ > 0.5). Because HRT is an efficient parameter in the operation of bioreactors, and the microorganism's exposure to contaminants is directly related to time. The results of other studies also showed that with the increase of HRT in the UASB and immobilized catalyzed packed bed reactor system, the removal efficiency of 4-CP and SCOD increased^25^. Based on these results, COD can be used as a good indicator for 4-CP monitoring in long-term exposures. In addition to microbial removal, it is also possible for 4-CP to be removed by physical methods due to the volatilization of 4-CP. The rate of this method increased during the turbulence of the reactor significantly. The average of physical removal of 4-CP is shown in Fig. 3 at different times.

As shown in Fig. 3, an increase in 4-CP concentration at the inlet of the reactor, especially at longer retention time, had a significant effect on the rate of physical removal (HRT = 96 h., R^2^ = 1, p < 0.05). Thus, physical removal has also been one of the affecting factors to the 4-CP decreasing concentration. The control reactor results also showed that non-biological processes such as aeration or bed adsorption contributed to 17% of the total removal of 4-CP. At low concentrations of 4-CP and SCOD (1–10 mg/l) due to the high removal efficiency, the role of non-biological processes was negligible. The results of the current study showed that by increasing the concentration of 4-CP in industrial wastewater, the rate of effective removal (biological removal) decreased, and ineffective removal rate (accumulation in biomass or release into the atmosphere during aeration) increased.

### Kinetic modeling for removal of 4-chlorophenol

Removal kinetics is one of the methods to predict the removal trend and reaction process. Removal kinetics were modeled by zero, one, pseudo-one, two, and pseudo-two equations to predict the mathematical behavior of the 4-CP removal by ALPBB. The best model for each initial concentration of 4-CP is shown in Table 5.

**Table 5:** The optimum kinetic model for removal 4-chlorophenol.

Optimum Model	Initial conc. (mg/L)	Function	SSE	R^2^	Adj-R^2^	RMSE
First-order	1	Y = 0.13 – 0.0167x	0.2921	0.836	0.782	0.312
Pseudo-first order	10	Y = 0.093e^0.0088x^	0.00055	0.953	0.938	0.0135
Pseudo-first order	100	Y = 0.0096e^0.0064x^	3.3 × 10^–7^	0.992	0.989	0.00033
Pseudo-first order	1000	Y = 0.001e^0.0028x^	6.9 × 10^–9^	0.886	0.847	4.8 × 10^–5^

According to Table 5, the best model to describe 4-CP removal by ALPBB at the concentration of 1 mg/L was the first-order equation, whereas, for 10, 100, and 1000 mg/L was the pseudo-first-order equation. Because the low concentration has less toxicity for the microbial population and the rate of 4-CP removal by physical processes is not significant. However, the removal rate of 4-CP at high concentrations by the physical process was significant. Due to the low performance of ALPBB to high 4-CP concentrations removal, and the emission of 4-CP to surrounding air by physical processes that can limit its application, it was recommended to combine this type of reactor to other methods such as biodegradation.

### Modeling 4-chlorophenol and SCOD removal in Air lift packed bed bioreactor

**Figure 4:**
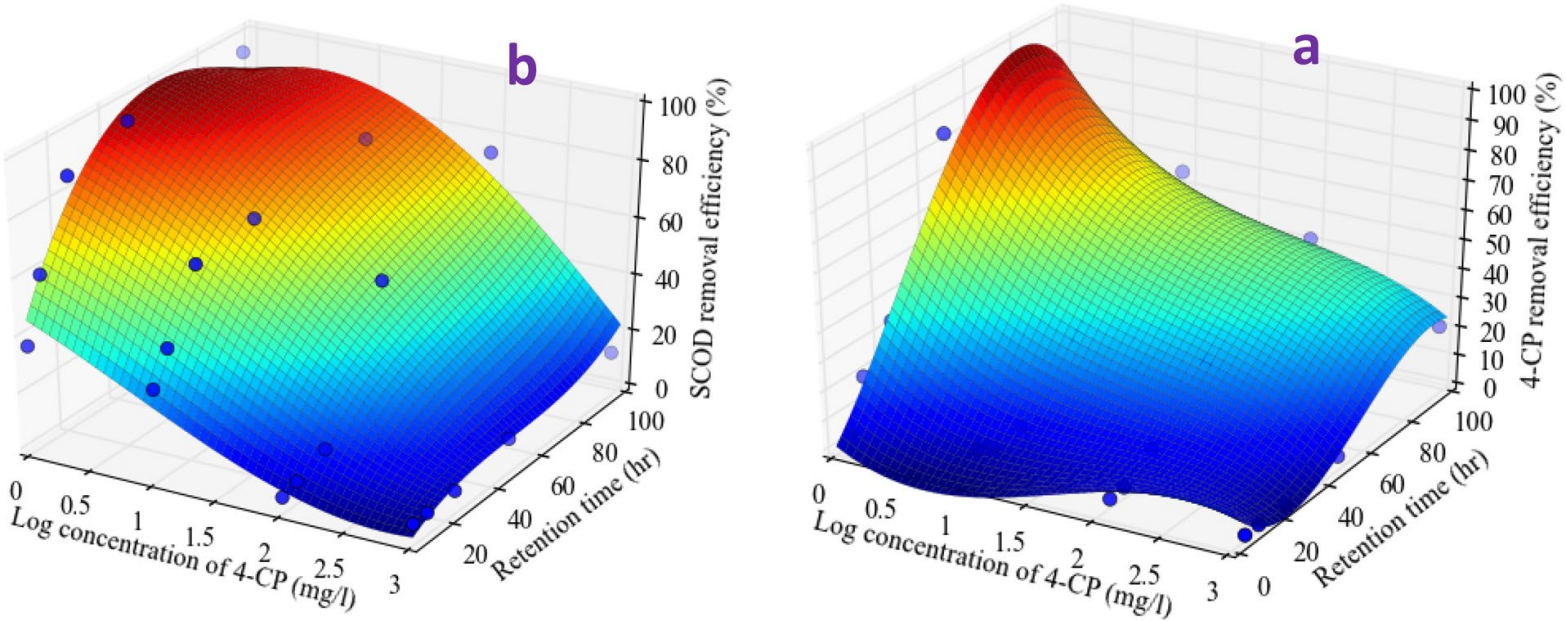
Profile for effects of input logarithmic concentration and the HRT on a) 4-chlorophenol and b) SCOD removal efficiency.

Simulation of the effect of concentration and HRT as independent variables (the constant of temperature and pH conditions) were modeled using Curve Expert software. The general equation of the model and the three-dimensional form of the mathematical model of 4-CP and SCOD removal are shown in Eqs. 3, 4, and Fig. 4, respectively.

3 $$ y = \frac{ - 20.1 - 7.1x1 + 3.6x2}{{1 + 2.7x1 + 0.02x2}} $$

4 $$ \begin{aligned} {\text{y}} & = 39.19 - 2.32{\text{X}}_{1} + 2.14{\text{X}}_{2} + 0.018{\text{X}}_{1}^{2} - 0.026{\text{X}}_{2}^{2} \\ & \quad - 0.000017{\text{X}}_{1}^{3} + 0.000091{\text{X}}_{2}^{3} + 0.0035{\text{X}}_{1} {\text{X}}_{2} \\ & \quad - 0.000005{\text{X}}_{1}^{2} {\text{X}}_{2} + 0.000012{\text{X}}_{1} {\text{X}}_{2}^{2} \\ \end{aligned} $$

The correlation coefficient of this model for the removal of 4-CP and SCOD was 0.93 and 0.96, respectively. Thus the current model is sufficient for the prediction of 4-CP and SCOD removal by ALPBB.

### The prediction of removal 4-chlorophenol by using ANN

**Figure 5:**
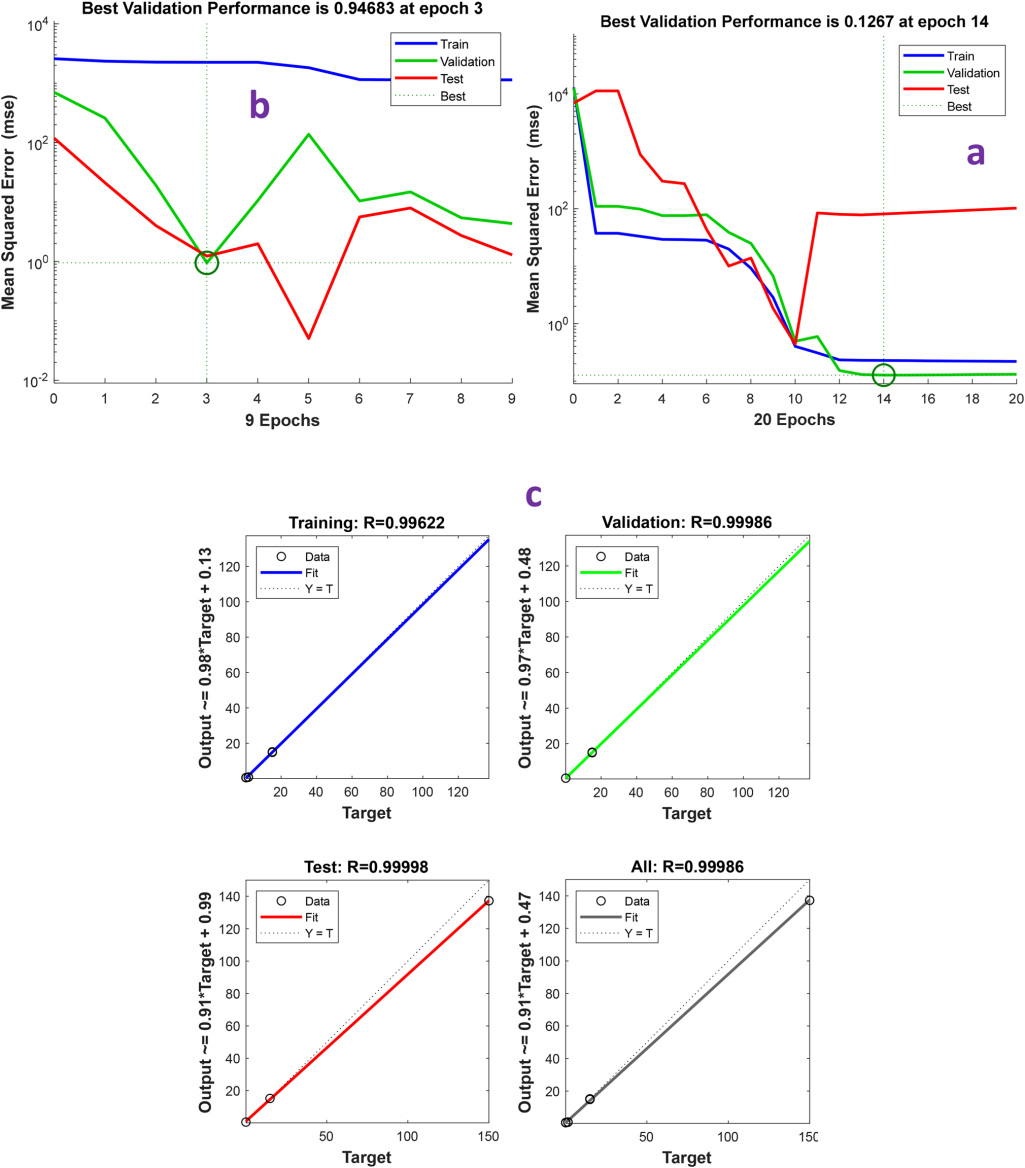
The prediction of removal of 4-chlorophenol using ANN. (**a**) Physical removal, (**b**) biological removal and (**c**) the regression coefficient of physical removal of 4-chlorophenol.

**Figure 6:**
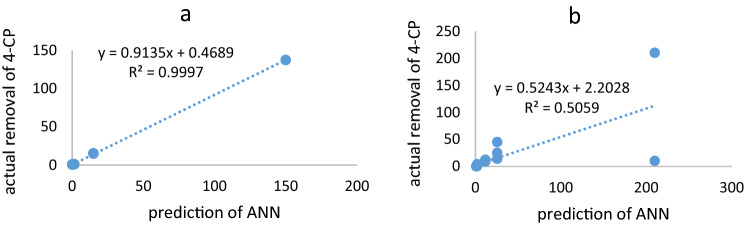
The correlation of actual removal of 4-chlorophenol and output of ANN. (**a**) Physical removal of 4-CP and (**b**) biological removal of 4-chlorophenol.

Due to the nonlinear relationship between 4-CP removal and the effect of exposure time variables and initial pollutant concentration, the ANN model was used to predict the 4-CP removal rate. The results of this modeling using the Levernberg-Marquantt algorithm and 2–6 neurons in the hidden layer showed that the best structure for physical and biological removal was 2:3:1 and 2:4:1, respectively, which is shown the lowest MSE and the highest correlation in Fig. 5. As shown in Fig. 6, the ANN output for 4-CP physical removal was highly correlated with the actual data (R^2^ = 0.9997). The minimum and maximum 4-CP removal were obtained by laboratory data and ANN at 1000 mg/l for 96 h., and 1 mg/L for 6 h., respectively. The correlation coefficient for actual biological elimination and ANN prediction was R^2^ = 0.5. Thus, ANN was highly capable of predicting 4-CP removal by physical methods, while its ability to biologically removal it was very low.

## Conclusion

The present study has investigated the ability of ALPBB to physical and biological removal of 4-CP and SCOD. To determine the best HRT, the ALPBB was operated at 5-time levels for different concentrations. According to the results, this reactor at 1 mg/l and 96 h. was able to remove near 85.3% of 4-CP from synthetic wastewater. Also, the removal efficiency depends on the power of wastewater and HRT, which decreases with increasing concentration and decreasing retention time.

The most important conclusions of this study are as follows:

- Also, by increasing the exposure time from 6 h. to 96 h., the removal efficiency increased at all concentrations. Moreover, with increasing 4-CP concentration, the removal efficiency decreased from 85 at 1 to 0.03% at 1000 mg/L. So that, there was positive correlation between COD removal and 4-CP increased (R^2^ = 0.5, HRT = 96 h.). Thus, in long-term exposure, the use of COD can be an appropriate indicator for monitoring 4-CP. The removal of 4-CP by physical methods at 1000 mg/L at 96 h. was 150 mg/L, which was much higher than other concentrations and lower retention times. Thus, the ALPBB did not have the desired performance in removing 4-CP at high concentrations, which is suggested this reactor modified.
- Correlation curves of 4-CP and SCOD based on the fit curve were 0.93 and 0.96, respectively. Also, the kinetics of 4-CP removal, at a concentration of 1 mg/L, follows the first-order equation, but at other concentrations follows the pseudo-first-order equation. However, the removal kinetics model of 4-CP depends on its concentration in the aqueous medium.
- 4-CP removal modeling by using the levernberg-marquantt algorithm has shown that 2:3:1 and 2:4:1 structure had the lowest MSE and the highest correlation (R^2^physical > 0.999 and R^2^biological > 0.71) was observed for 4-CP removal. Also, the ANN obtained output of 4-CP physical removal was highly correlated with the actual data (R^2^ = 0.9997). As such, ANN has a high ability to predict 4-CP removal by using the physical method, while its ability to predict biological removal is low.
- In brief, this bioreactor is an effective method for biodegradation of 4-CP at low concentrations. Whereas, it is essential to modify this reactor for removal of high concentration of 4-CP.
